# The Silent Killer Seeks Young Blood: A Case Report on the Perioperative Anesthetic Management of a Paraganglionoma in Pregnancy

**DOI:** 10.7759/cureus.44025

**Published:** 2023-08-24

**Authors:** Samantha Ballard, Johannes J Le Roux, Koji Wakabayashi, Hendrik C Labuschagne, Zainub Jooma

**Affiliations:** 1 Department of Anesthesia, Charlotte Maxeke Johannesburg Academic Hospital, Johannesburg, ZAF; 2 Department of Radiology, Charlotte Maxeke Johannesburg Academic Hospital, Johannesburg, ZAF; 3 Anesthesia and Critical Care, Charlotte Maxeke Johannesburg Academic Hospital, Johannesburg, ZAF; 4 Department of Anesthesia, University of the Witwatersrand, Johannesburg, Johannesburg, ZAF

**Keywords:** pregnancy, caesarean section, general anesthesia, pheochromocytoma, paraganglionoma

## Abstract

Pheochromocytomas and paraganglionomas are rare neuroendocrine tumors. The multisystem effects of these tumors on the pregnant woman and fetus, the timing of surgery in relation to the pregnancy, and the pharmacological treatment have several anesthetic implications. Case reports on elective cesarean section followed by postpartum resection of the tumor are scarce. A case is presented of a 31-year-old nulliparous female where an antenatal diagnosis of a paraganglionoma was made at 19 weeks gestation for whom an elective cesarean section was performed at 31 weeks gestation under graded lumbar epidural anesthesia, followed by an elective open surgical removal of the tumor six weeks postpartum.

## Introduction

Pheochromocytoma (PHEO) and paraganglionoma (PGL), collectively known as pheochromocytoma and/or paraganglionoma (PPGL), are rare neuroendocrine tumors originating from chromaffin cells within the autonomic nervous system [1]. The majority (80-85%) of chromaffin cell tumors are PHEO, which arise from within the adrenal medulla. The remaining 15-20% are PGL and stem from extra-adrenal chromaffin cells located in the sympathetic paravertebral ganglia [2]. Globally the incidence of PPGL in adult patients with hypertension is estimated to be 0.1-0.6% [2]. PPGL in pregnancy is rare and is estimated to occur in 0.0002% of pregnancies [3]. Fewer than 300 PPGLs have been described in the literature in the parturient population, with the majority being diagnosed in the postpartum period. Thirty-eight cases of PGL during pregnancy have been documented since 1980 [4].

When hypertension, often referred to as the "silent killer," is diagnosed in pregnancy before 20 weeks gestation, PPGL should be actively excluded as part of the workup for secondary causes of hypertension [1]. The classically described symptoms of paroxysmal headaches, diaphoresis, palpitations, and tachycardia are less common in pregnancy, challenging the consideration for PPGL [1, 5]. Early diagnosis, localization of the tumor, and initiation of treatment are central to constructing the multidisciplinary perioperative management plan to ensure good maternal and fetal outcomes.

A case is presented of a 31-year-old female where an antenatal diagnosis of PGL was made for whom an elective cesarean resection was performed at 31 weeks gestation, followed by an elective open surgical removal of the tumor six weeks post-partum.

## Case presentation

A 31-year-old, previously healthy nulliparous female, presented to the antenatal clinic at 19 weeks gestation with intermittent flushing, sweating, and headache. Her symptoms had started four weeks before her presentation to the antenatal clinic. She was not known with any comorbidities, had sober habits, was a non-smoker, did not report any allergies, and had no significant family history of multiple endocrine neoplasia (MEN). On presentation, her blood pressure (BP) was 152/89 mmHg, with no proteinuria noted on urinalysis. A second elevated BP (152/95 mmHg) was confirmed on a follow-up to the antenatal clinic, and she was diagnosed with hypertension. A workup for secondary causes of hypertension was commenced.

**Figure 1:**
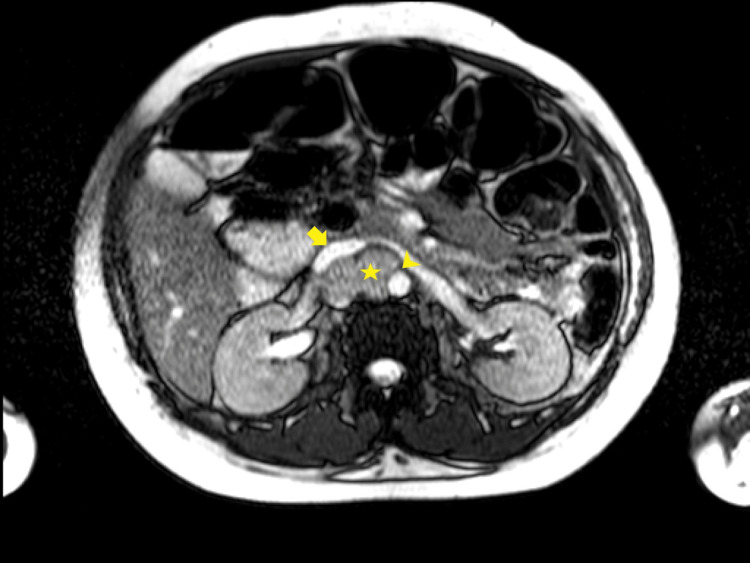
Axial T2 NFS shows an intermediate-to-high signal bilobed right para-aortic mass (star), with encasement (but not invasion) of the abdominal aorta (arrowhead) and more than 180-degree contact with the inferior vena cava (arrow). The lack of clear separation planes from these vital vascular structures requires exceptional surgical prowess given the increased risk of haemorrhage upon excision.

**Figure 2:**
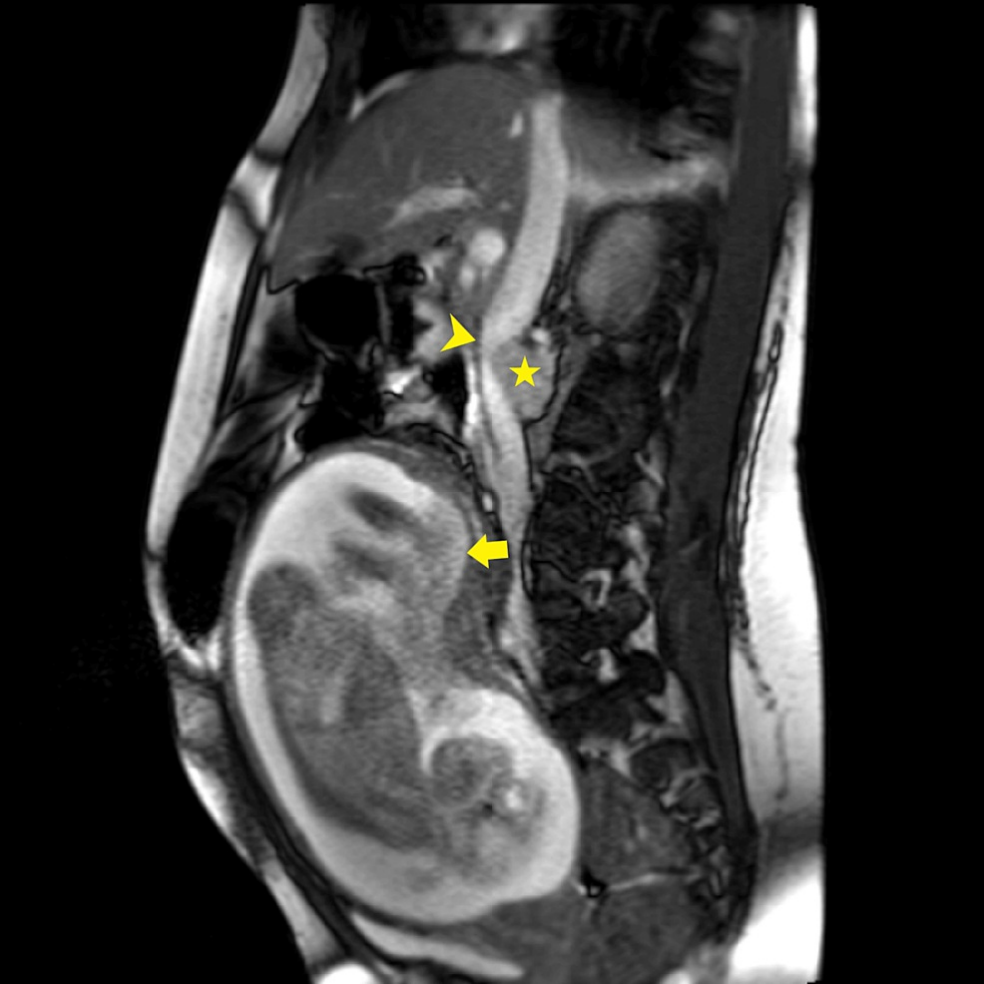
Sagittal T2 NFS exemplifying a bright, well-circumscribed anterior paraspinal mass (star) anteriorly displacing the dorsal margin of the inferior vena cava (arrowhead). Concurrent intra-uterine pregnancy with high posterior position of the placenta (arrow).

**Figure 3:**
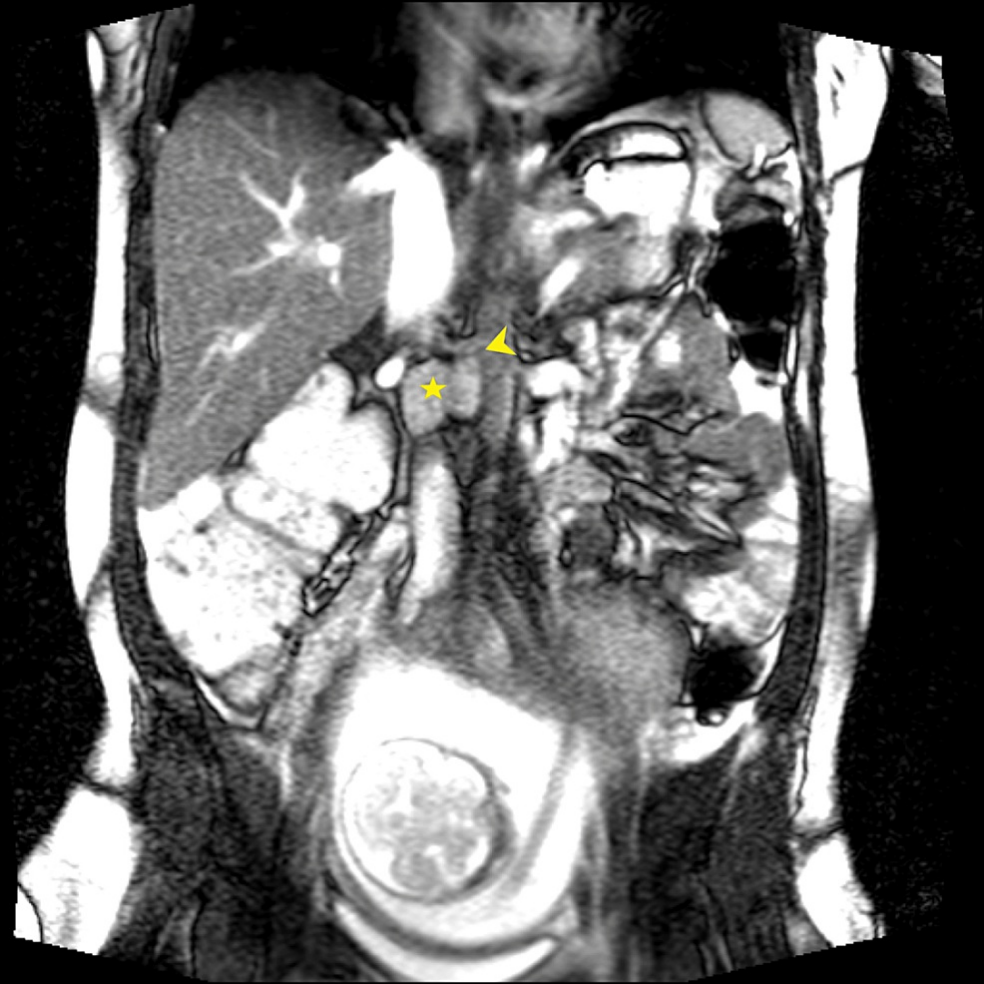
Coronal T2 NFS demonstrates an intermediate signal lobulated encapsulated mass (star) straddled by the right renal artery-aortic bifurcation (arrowhead).

A 24-hour urine excretion of fractionated metanephrines was performed, which confirmed a raised vanillylmandelic acid (VMA) excretion of 85 µmol/24 h (normal 7.0-33.0 µmol/24 h) and a raised VMA to creatinine ratio of 8.4 (normal 1.6-4.7). Metanephrine excretion was normal at 290 nmol/24 h (normal 152-913 nmol/24 h). Normetanephrine excretion was significantly raised at 13,550 nmol/24 h (normal 606-2,288 nmol/24 h), and the normetanephrine to creatine ratio was also significantly raised at 1,338.9 (normal 57-234). She was diagnosed with a PPGL and started on doxazosin 4 mg orally twice daily. Evaluation of the fetus by ultrasonography did not reveal any abnormalities and confirmed adequate placental perfusion. Magnetic resonance imaging (MRI) was done to confirm the location of the PPGL. A well-defined para-aortic mass measuring 2.8 cm x 4.3 cm x 3.7 cm at the level of the L2 vertebra was noted (Figures 1-3).

**Figure 4:**
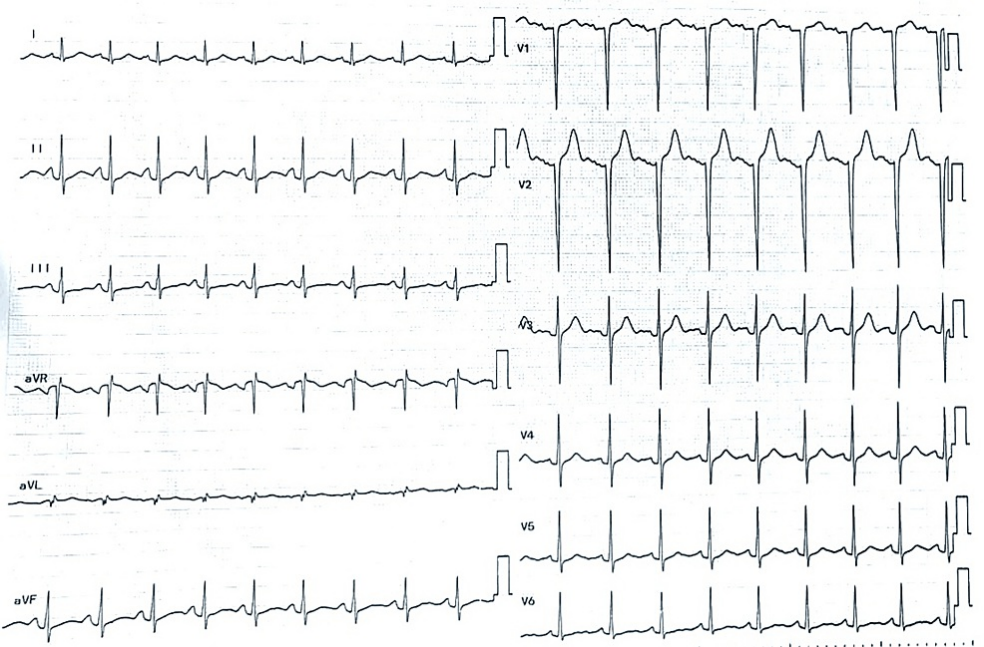
Electrocardiogram with features suggestive of left ventricular hypertrophy.

The electrocardiogram (ECG) showed features suggestive of left ventricular hypertrophy (LVH) (Figure 4), which was confirmed by transthoracic echocardiography (TTE). No other abnormalities were noted on TTE. Apart from LVH, no other end-organ dysfunction was noted in this patient.

A multi-disciplinary team was consulted, including obstetricians, endocrinologists, intensivists, and the patient. A decision was made to perform an elective cesarean section at 34 weeks gestation. The tumor would be surgically removed postpartum as the complex location of the tumor could potentially pose a risk to the fetal well-being, and this was a desired pregnancy for the patient.

At 25 weeks gestation on follow-up at the antenatal clinic, the patient was noted to have a systolic BP of 210/122 mmHg despite oral antihypertensive treatment. She was admitted to a high dependency unit (HDU) for BP monitoring and control, and an intravenous (IV) labetalol infusion was started. Her oral medication at this time included 8 mg doxazocin, 3.125 mg carvedilol bd, and 5 mg amlodipine bd. She was discharged from the HDU after five days; however, uncontrolled BP persisted, and the patient was readmitted to the HDU for BP control at 30 weeks gestation. Her BP was invasively monitored via an arterial line that was inserted into her right radial artery, her oral medications were stopped, and an IV labetalol infusion was reinitiated to achieve target BP. A decision was made to perform an elective cesarean section at 31 weeks gestation after the administration of intramuscular steroids to achieve fetal lung maturity.

In theatre, a left radial arterial line and a right femoral vein central line were inserted under local anesthesia using ultrasound guidance. A graded lumbar epidural (LEA) was performed in the sitting position at vertebral level L3/L4 using a loss-of-resistance to saline technique. The epidural catheter was secured 5 cm into the epidural space. A test dose of 3 mL of 1.5% lignocaine was administered. No adrenaline was added to the test dose to avoid a potential hypertensive response in the event of the epidural catheter inadvertently being inserted into a vessel. A T4-L1 sensory anesthetic level was obtained with 15 mL of 0.5% bupivacaine, administered in 5 mL boluses, 5 minutes apart. A lower-segment cesarean section was performed by a senior obstetrician at our institute. The lumbar epidural was again bolused after 40 minutes, with 5 mL of 0.5% bupivacaine. No systemic analgesia was administered intra-operatively. The patient's starting BP was 137/86 mmHg. During uterine manipulation before delivering the fetus, the patient's BP increased to 210/132 mmHg, and 1 mg nitroglycerine was administered intravenously, after which her BP normalized to 122/68 mmHg. A live baby weighing 1,850 g was delivered, with APGAR scores of 8/10 and 10/10 at 1 and 5 minutes, respectively. The uterus was not externalized after delivery of the fetus to avoid potential traction or compression on the tumor with the subsequent release of catecholamines. No adverse effects were noted when IV oxytocin was administered. Prior to skin closure, the patient had another increase in her BP (185/122 mmHg), and a bolus of 5 mg labetalol was administered intravenously, after which her BP decreased to 134/78 mmHg. The duration of surgery was 42 minutes, and intra-operative blood loss was estimated at 250 mL. 

Post-operatively, she was transferred to an HDU where her BP was invasively monitored. Oral anti-hypertensives were recommenced, and adequate BP control was achieved. The epidural catheter was kept in situ for two days, and intrathecal morphine was administered to provide analgesia. She was discharged on day three post-surgery to the maternity unit as she remained hemodynamically stable. The reduction in her intra-abdominal pressure after delivery of the fetus with presumed less mechanical pressure on the tumor did not lead to a reduction in her antihypertensive drug requirements.

**Figure 5:**
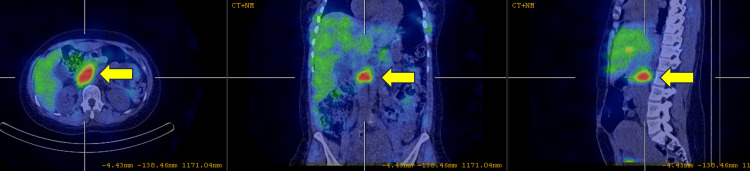
An iodine meta-iodobenzylguanidine (MIBG) scintigraphy scan confirming tracer uptake to a soft tissue mass at the level of the L2 vertebra.

One week postpartum, an iodine meta-iodobenzylguanidine (MIBG) scintigraphy scan was performed, which confirmed focal uptake of the tracer to an aorto-caval soft tissue mass at the level of the L2 vertebra (Figure 5). There was no evidence of distant metastases.

Elective removal of the PPGL was delayed for six weeks postpartum to allow for uterine involution. The patient underwent an uneventful open resection of the tumor under a general anesthetic. Postoperatively, she was observed for two days in an HDU where she remained hemodynamically stable off all anti-hypertensive medications. Her urine catecholamine levels were tested after two days, which were within the normal limits, after which she was discharged from the HDU to a ward.

## Discussion

Although PPGL in pregnancy is rare, if unrecognized and/or untreated, it may result in a life-threatening hypertensive crisis precipitated by mechanical factors, such as the enlarging uterus, fetal movement, uterine contractions, vaginal delivery, and general anesthesia [6]. Elevated catecholamines secondary to PPGL result in adverse consequences for the pregnant woman and fetus [7]. The hypertensive crisis can result in hemorrhage and organ infarction, cardiac dysrhythmias, congestive cardiac failure, uteroplacental insufficiency, and death. Uteroplacental insufficiency translates into intrauterine growth restriction, fetal hypoxia, and death [6]. A systematic review by Bancos et al. [4] concluded that severe maternal complications and maternal death occurred in 7% of pregnancies with untreated PPGL. The main causes of maternal mortality include dysrhythmias, cerebrovascular incidents, and pulmonary edema [5]. The fetal mortality rate has been estimated at 26% in undiagnosed PPGL and 11% in diagnosed cases [4].

Recognizing PPGL in pregnancy is challenged by the low incidence of classic symptoms, such as paroxysmal headaches, diaphoresis, palpitations, and tachycardia [1,5]. Symptoms may appear for the first time during pregnancy or worsen during pregnancy, because of increased vascularity of the tumor and mechanical factors, such as pressure from the enlarging uterus, stimulating catecholamine secretion [6]. PPGL can express the luteinizing hormone/chorionic gonadotropin receptor (LHCGR), resulting in faster tumor growth in pregnancy [3]. Persistent or paroxysmal hypertension is the most common clinical sign of PPGL in pregnancy as only 5-15% of patients are normotensive when a PPGL is diagnosed antenatally [8]. Hypertension during pregnancy is often attributed to gestational hypertension or pre-eclampsia, making the diagnosis of a PPGL more difficult [7]. In up to 50% of cases, the diagnosis of a PPGL is only made during the postpartum period [5]. When hypertension, often referred to as the "silent killer," is diagnosed in pregnancy before 20 weeks gestation, PPGL should be actively excluded as part of the workup for secondary causes of hypertension [1].

Antenatal diagnosis of PPGL and localization of PPGL is necessary to formulate a multidisciplinary peripartum management plan where the aim is to decrease maternal and fetal morbidity and mortality [1]. PPGL is confirmed biochemically by elevated urinary levels of catecholamines and their metabolites (metanephrines, norepinephrine, and vanillyl mandelic acid) or raised plasma catecholamines [7]. Obtaining a detailed drug history is essential as tricyclic antidepressants can lead to a false-positive result [7]. There is no change in the diagnostic range of urinary catecholamines during pregnancy; thus, the diagnosis will not be obscured [9].

From an imaging standpoint, a multipronged approach harnessing ultrasound, CT, MRI, and nuclear imaging is often employed [10-12]. Although the radiological features of PGLs are varied, the best diagnostic clue to a PGL is alluded to by a T2-hyperintense (classically “light-bulb” bright) mass along the expected location of the sympathetic chain on MRI [10,11]. In this instance, Figures 1-2 exemplify the hyperintense appearance of the well-encapsulated mass along the abdominal sympathetic chain. Figures 1-2, and in particular Figure 3, show the high-risk relationship of the PGL with critical vascular structures, necessitating meticulous antecedent surgical planning and intraoperative technique. More than 85% of extra-adrenal sympathetic chain PGLs are found caudal to the diaphragm within the abdomen, and despite sometimes being detected incidentally, localizing these masses on imaging in the setting of the appropriate clinical history and supporting biochemical tests is paramount to the successful further management of these individuals [10,11]. Ultrasound can sometimes be of clinical utility in finding suspected PGLs, but MRI and CT are typically needed for exact delineation prior to surgical management [11]. MRI provides superior imaging and is preferable to ultrasonography as a modality to localize the tumor in pregnancy while avoiding radiation exposure to the fetus [5,6]. Ultrasonography has a reported sensitivity of 89-97% for the detection of adrenal masses, while MRI additionally allows for the identification of extra-adrenal tumors [6]. Functional nuclear imaging often adds value and specificity with Ga-68 DOTATATE (somatostatin analog tagged radiotracer) binding to tumors with somatostatin receptors (SSTR2), of which PGLs are a prime example [12,13]. Although Ga-68 DOTATATE has been reported to have a superior sensitivity and imaging resolution to 131I-MIBG, 131I-MIBG (as a guanethidine analog bound to a tracer) is available at our institution and relies on the PGL storing the MIBG, allowing for detection of increased tracer uptake within the mass [10,12]. MIBG scintigraphy has a sensitivity of 77% and may be considered for tumor localization postpartum. Should MIBG scintigraphy be required intrapartum, the radiation risk to the fetus should be weighed against the maternal benefit obtained from the investigation [6].

Once the diagnosis of PPGL in pregnancy has been made, pregnant patients should be managed by a multidisciplinary team, consisting of obstetricians, neonatologists, maternal-fetal medicine specialists, endocrinologists, anesthesiologists, intensivists, and vascular surgeons with experience in the management of PPGL.

Medical management includes alpha-adrenergic blockade (phenoxybenzamine, prazosin, or doxazosin) to manage hypertension and beta-adrenergic blockade (carvedilol, metoprolol) to treat dysrhythmias and tachycardia [5]. Alpha-blockers that may be used are phenoxybenzamine (non-selective) or doxazosin (selective), and both achieve similar ranges of normotension [7]. The goal of medical treatment of PPGL in pregnancy is to achieve a low normal systolic BP for a patient's age, typically 90-100 mmHg [3]. The alpha-adrenergic blockade can be intensified at 36 weeks to prepare for delivery while still maintaining the goal systolic BP of 90-100 mmHg [3]. The target heart rate should be less than 90 beats per minute [3]. Adequate alpha-adrenergic blockade generally requires 10-14 days of treatment and should be initiated at least one week before beta-adrenergic blockade [3,6,7]. The addition of calcium channel blockers (nifedipine, amlodipine) may be considered to control resistant hypertension and should be started after 2-3 weeks of alpha-adrenergic blockade [3]. The use of alpha-adrenergic blockers, beta-adrenergic blockers, and calcium channel blockers in pregnancy may be considered after the risks and benefits have been discussed with the patient, and a shared decision regarding its use is made between the medical team and the patient [3]. Phenoxybenzamine crosses the placenta and may result in hypotension in the newborn after delivery. Maternal doxazosin treatment has not been associated with neonatal hypotension [14]. Atenolol may cause low birth weight and propranolol may result in neonatal hypoglycemia and bradycardia [3]. Neonatal monitoring following delivery is advised for 48-72 hours should the fetus have been exposed to any beta-adrenergic blocker [3]. Breastfeeding appears to be safe with both phenoxybenzamine and doxazosin [7]. Certain medications may trigger catecholamine release and precipitate a hypertensive crisis and should be avoided in a pregnancy associated with PPGL, namely, metoclopramide, steroids, and sympathomimetics [15].

Surgical resection, either laparoscopically or open, is the only curative treatment for PPGL. The timing of such surgery remains controversial for PPGLs diagnosed in pregnancy [1,3,4,6,9]. The optimal timing of surgery in relation to the pregnancy depends on the gestational age at which the diagnosis is made, the severity of the disease, how well the patient responds to medical therapy, tolerance to medical therapy, maternal complications, or end-organ dysfunction secondary to persistent hypertension and fetal wellbeing [3]. It has been postulated that, should pharmacological blockade be achieved before 23 weeks gestation, surgical resection should be considered, ideally after the first trimester [3].

If the pregnancy is more than 24 weeks gestation, surgery should be delayed if possible until delivery of the fetus, either via a cesarean when fetal maturity has been reached or via vaginal delivery in selected cases [3,6,16]. Surgical removal of PPGL in the third trimester should be avoided as the uterine size might limit visibility in the pelvis and abdomen [3]. A systematic review by Langton et al. [17] noted twice as many women carried pregnancies to term when tumors were resected antepartum (81%) compared with postpartum (41%). Minimally invasive surgical techniques, such as robotic-assisted surgery, can be considered as it improves dexterity in small spaces, which may be helpful for the removal of large PGL in the retroperitoneum or surgery in an obese patient [3].

Indications for early delivery include the presence of IUGR, fetal distress, and uncontrolled maternal BP despite optimal medical therapy [18]. Two recent systematic reviews by Bancos et al. [4] and Langton et al. [17] concluded that more than 66% of pregnancies associated with PPGL were delivered by cesarean section. Patients with large tumors, particularly within the abdomen and pelvis, and with high catecholamine levels should preferably deliver via cesarean section [4]. Successful vaginal delivery can be achieved with adequate alpha-blockade in selected cases [4,17,19]. To minimize the risk of tumor stimulation from raised intra-abdominal pressure during maternal pushing, an elective epidural and passive second stage may be prudent [19]. Pregnant women in whom a vaginal delivery may be considered include patients with small tumors, PGL located outside the abdomen and pelvis, and multiparous women who deliver more quickly [3].

The perioperative anesthetic plan should include ensuring fetal lung maturity with preoperative systemic corticosteroids (should surgery be performed after fetal viability), adequate peri-operative alpha-adrenergic blockade, prevention of surgical stress response during surgery, anticipating and managing fluctuations in BP, maintaining uteroplacental blood supply, and monitoring both baby and mother in an HDU postoperatively [3,6,9]. Performing a cesarean section in a patient with a PPGL poses a challenge to the anesthetist, as manipulation of the uterus can result in catecholamines being secreted, leading to intra-operative hypertension, resulting in increased perinatal and maternal morbidity and mortality [3].

The anesthetic should be tailored to account for these challenges. Sympathomimetic drugs and events that cause stimulation of the sympathetic nervous system should be avoided. Intra-operative monitoring should include invasive BP monitoring to detect catecholamine-induced cardiovascular changes. If a GA is planned, blunting of the intubation response must be considered as laryngoscopy and tracheal intubation can trigger catecholamine release and precipitate a hypertensive crisis. Rapid-acting opioids (remifentanil), nitroglycerine, short-acting beta-adrenergic blockers (esmolol and labetalol), and magnesium sulfate are useful adjuncts [3,20]. All induction agents are safe, except for ketamine, which may stimulate the sympathetic nervous system [20]. It is also recommended to avoid non-depolarizing muscle relaxants with vagolytic or histamine-releasing properties [20]. Maintenance of anesthesia may be achieved with the administration of volatile anesthetic agents, such as isoflurane and sevoflurane, avoiding halothane as it sensitizes the myocardium to circulating catecholamines, as well as desflurane due to the catecholamine release it causes when administered rapidly [20].

Neuraxial anesthesia in the form of a graded lumbar epidural has been successfully used to achieve these goals [20]. To manage a hypertensive crisis, it is recommended that IV phenoxybenzamine or phentolamine be available. Intravenous labetalol is the appropriate alternative if IV alpha-blockade is not available [6]. Intravenous nicardipine may be considered should IV alpha-blockade be ineffective [3]. Intravenous hydralazine should be avoided as it may cause acute coronary syndrome in patients with PPGL [3].

Elective termination of pregnancy should be considered when the PGL is deemed life-threatening, which may be the case when the PGL is located in the pelvis or genitourinary tract in close proximity to the enlarging uterus [3]. Persistent hypertension, despite optimal medical management, is an indication for surgical resection of the tumor, but the surgery might be high risk based on the location and size of the tumor [3]. The patient should be assessed by the multidisciplinary team and the risk to both the pregnant woman and fetus established. Support should be provided throughout the peripartum period to the pregnant woman, with a focus on psychological support especially when elective termination of pregnancy is considered [3].

PPGLs in pregnancy are associated with germline pathogenic variants (GPV). Bancos et al. [4] concluded that GPV were present in 66% of patients with a peripartum diagnosis of PPGL, including variations in succinate dehydrogenase complex iron sulfur subunit B, ret proto-oncogene associated with MEN syndrome, and the Von-Hippel-Lindau (VHL) gene. All patients with PPGL, diagnosed in the peripartum period who do not have a known GPV for PPGL or have not been previously tested, should be offered genetic counseling and testing [3].

The strengths of this report are discussing PGL in pregnancy as an uncommon cause of secondary hypertension, and detailing the perioperative management of PGL with a focus on the intra-operative anesthetic management of a cesarean section. This report is limited by the lack of data comparing concurrent surgical resection of the tumor during cesarean section versus delayed postpartum resection.

## Conclusions

The goals in the management of PPGL in pregnancy encompass early diagnosis and initiation of medical therapy, avoidance of peripartum hypertension, and definitive surgical resection of the tumor. The timing of surgical resection will depend on the gestational age at which the diagnosis was made and the presence of maternal end-organ dysfunction. When the tumor is still present, cesarean section is the preferred mode of delivery, although vaginal delivery can be considered in select cases. This case report illustrates how a graded LEA technique was used as the sole anesthetic technique to provide anesthesia for an elective cesarean section in a patient with a PGL, followed by postpartum resection of the tumor six weeks later.
